# Evaluation of Vaccination Strategy Against Rabies in Hong Kong Macaques

**DOI:** 10.3389/fvets.2022.859338

**Published:** 2022-03-21

**Authors:** Paolo Martelli, Sophie St-Hilaire, Wai-Suk Hui, Karthiyani Krishnasamy, Ioannis Magouras, Omid Nekouei

**Affiliations:** ^1^Ocean Park Corporation Hong Kong, Aberdeen, Hong Kong SAR, China; ^2^Department of Infectious Diseases and Public Health, Jockey Club of Veterinary Medicine and Life Sciences, City University of Hong Kong, Kowloon, Hong Kong SAR, China

**Keywords:** rabies, vaccination, wildlife, macaque, immunogenecity, ELISA

## Abstract

The objectives of this study were to assess the serological response to rabies vaccination in Hong Kong macaques and provide evidence-based recommendations for the vaccination interval implemented by the Government of Hong Kong. An inactivated rabies vaccine was administered subcutaneously to captured macaques under a mass sterilization program in Hong Kong. Blood samples from the animals were collected in a 2015 field survey and stored in −80°C freezer. In 2021, the frozen sera from vaccinated animals were prepared and tested for antibodies against the rabies virus using a commercial blocking enzyme-linked immunosorbent assay (ELISA) test. Sixty-five samples were available from the vaccinated macaques that had received at least one dose of the vaccine between 2008 and 2015. The interval between the first vaccination and blood sampling ranged from 21 to 2,779 days (median: 990). Only five macaques had a second vaccination record at the sampling time, all with high antibody levels. Among the remaining macaques, 77% (46/60) were positive for rabies antibodies. No specific association was observed between the post-vaccination period and the antibody titer of these macaques, and no adverse reactions were reported. Although the precise level of protection against a potential challenge with the rabies virus cannot be ascertained, the vaccination elicited rabies antibodies in 87% (21/24) of the macaques tested within 2.5 years of their first vaccination. Our findings indicate the potential benefits of the current vaccination strategy to protect the population from rabies and consequential mandatory culling of all macaques if a natural infection occurs.

## Introduction

Rabies is a fatal zoonotic disease that can affect all mammals. It is estimated to kill 59,000 people every year across the world, with 95% of cases reported from Africa and Asia (1). In Hong Kong, the last indigenous human case of rabies was reported in 1981, and two imported cases were detected in 2001 and 2014. The last report of rabies in an animal (a dog) in Hong Kong dates back to 1987. However, rabies remains a significant public health concern in China, especially close to the southern borders with Hong Kong, leading to hundreds of human deaths every year (2, 3).

**Figure 1:**
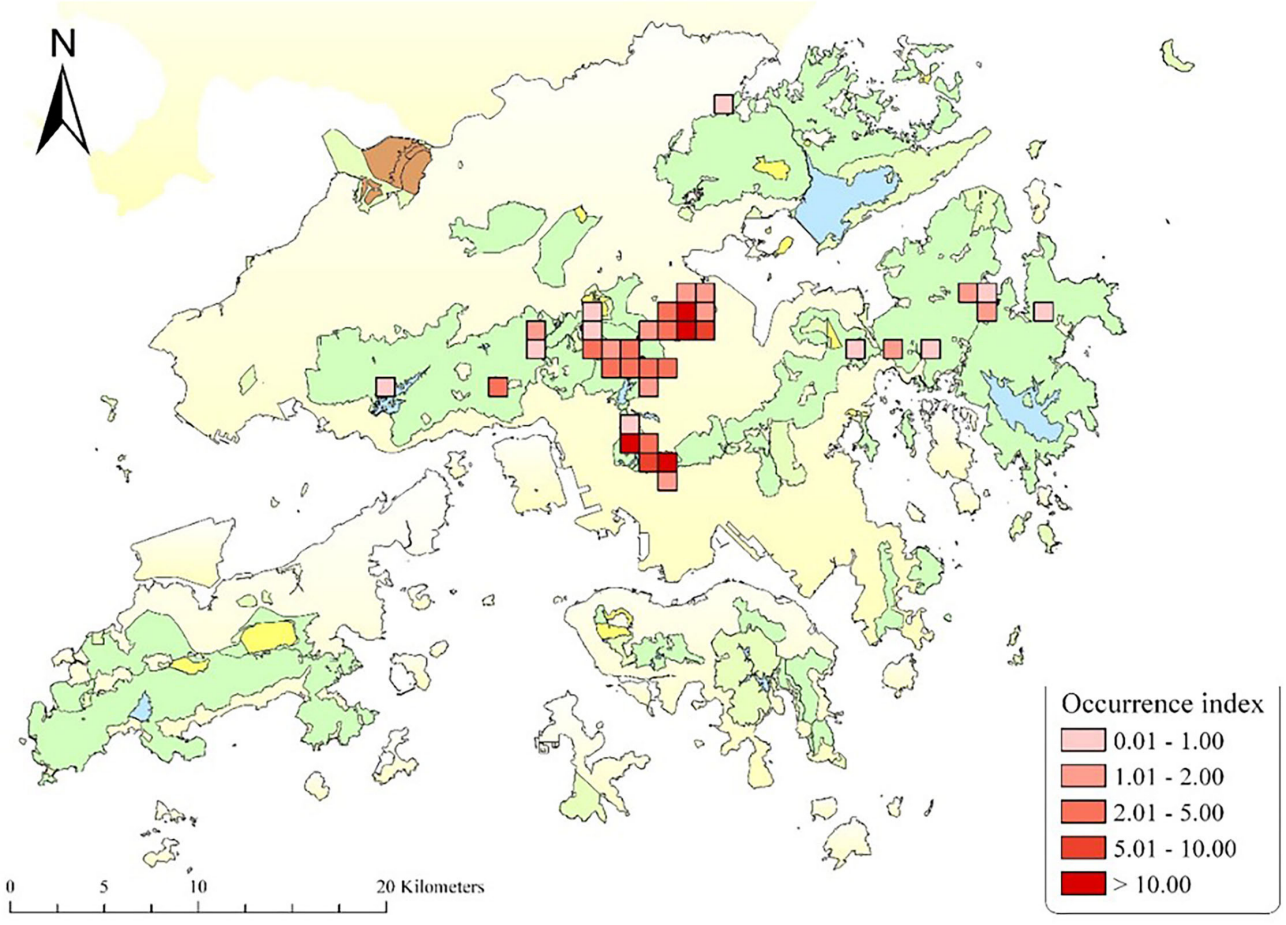
Map of Hong Kong displaying the trapping sites of the macaques for the contraceptive program, between 2009 and 2021. The Occurrence Index is defined as the number of photographs taken divided by the total amount of trapping effort in 100 camera working days [detailed in (4)]. The map is provided by the Agriculture, Fisheries, and Conservation Department, The Government of the Hong Kong SAR.

There are ~1,800 wild monkeys in Hong Kong, distributed in 30 social troops, mainly inhabiting Kam Shan, Lion Rock, and Shing Mun Country Parks (Figure 1). The majority of them are considered hybrids of Rhesus Macaque (*Macaca mulatta*) and Long-tailed Macaque (*M. fascicularis*) (5). Despite a ban on feeding wild animals in Hong Kong, the macaques are regularly fed by hikers and tourists and, therefore, come in close contact with humans and their pets, feral dogs, as well as other wild animals within their habitat. There have been reports of occasional aggressive encounters between the macaques and residents in Hong Kong (6). Although rabies has never been reported in Hong Kong macaques, all primates are susceptible to rabies (7), and there are several reports of rabies in non-human primates in other regions where the pathogen is endemic, leading to human exposures and cases of the disease (7–11). The aggressive form of rabies is rarely seen in non-human primates, so it is difficult to differentiate the signs of disease from natural biting patterns in monkeys (12), possibly resulting in the underreporting of rabies in non-human primates (9, 13).

In 2000, the Government of the Hong Kong SAR (Agriculture, Fisheries and Conservation Department) initiated a pilot project for the mass capture and sterilization of macaques. The pilot project mainly included small-scale population studies, public consultation, and limited volume of capture and strelization. Since 2009, that project has substantially been expanded by adding more financial support and resources, expertise, and it is still ongoing (14). All captured macaques under the sterilization program (laparoscopic tubectomy) have been vaccinated against rabies using Rabisin® (Merial, Boehringer Ingelheim Animal Health) and received tag IDs as per the directive of The Rabies Ordinance (Cap. 421) of the government. Monkeys that were recaptured 2.5 years or longer after their last vaccination received a booster vaccination.

Although this vaccination protocol has been in place for over 20 years, the immune status of the macaques against the rabies virus has not been evaluated. The objectives of this study were to assess the serological response to rabies vaccination in the population of macaques in Hong Kong, and to provide evidence-based recommendations for the vaccination interval implemented by the Government of the Hong Kong SAR for this wild animal population.

## Method

**Figure 2:**
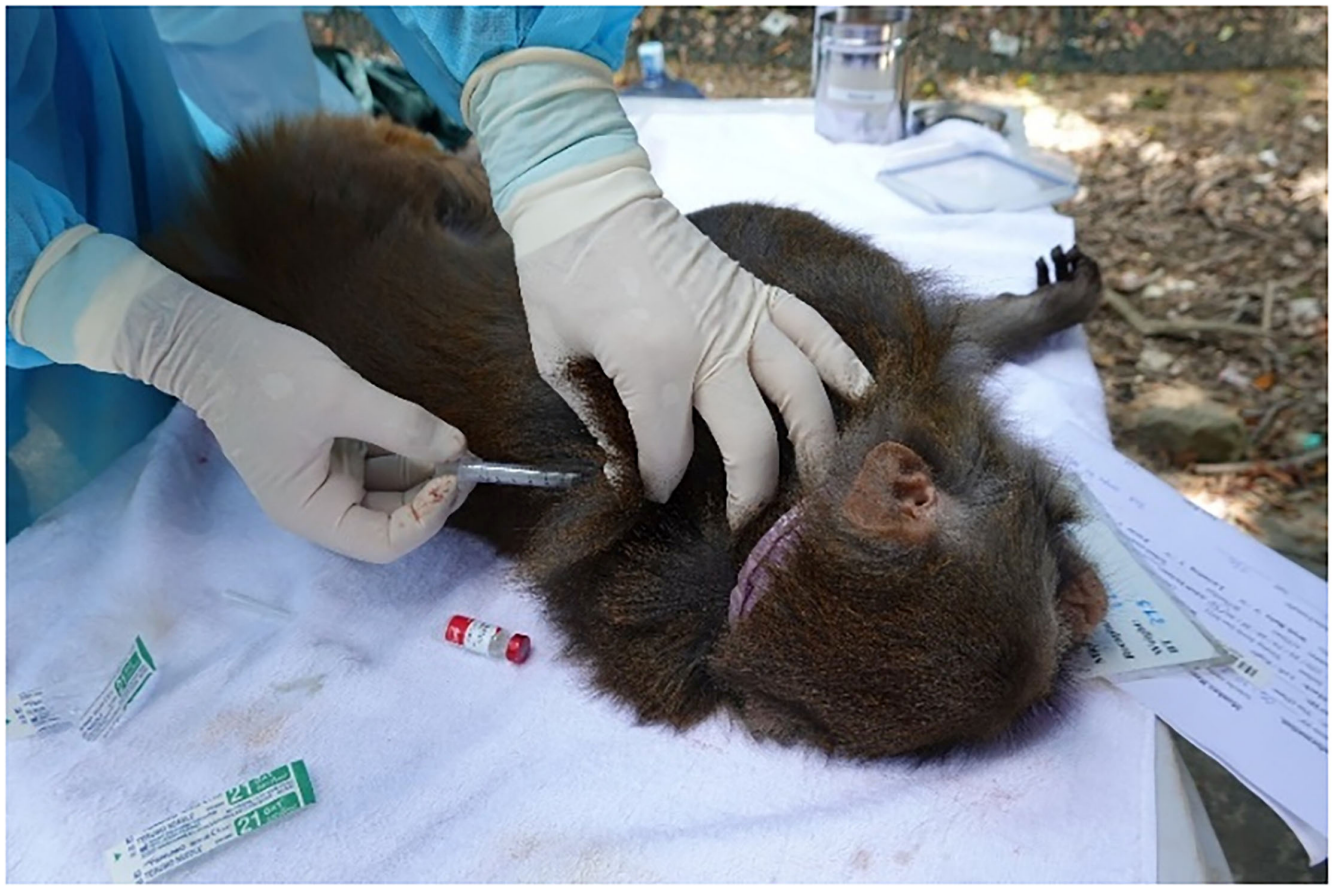
Subcutaneous injection of the rabies vaccine (1 ml) in the interscapular space of a Hong Kong macaque under anesthesia, using a 3 ml syringe and a 23-gauge needle.

As a part of the sterilization program of macaques (14), a population survey and trap habituation were carried out between 2009 and 2021. Groups of 15 to 129 macaques were captured in communal traps. Trapping was carried out 10 to 24 times a year. A mobile partition inside the communal trap was used to repeatedly drive smaller groups of 1–4 animals into a small squeeze cage, where they were injected with a combination of anesthetic drugs consisting of 0.05 mg/kg medetomidine (Dorbene®, Laboratories Syva, Spain) and 5 mg/kg ketamine (Ketamine®, Alfasan International BV, Holland). Rabies vaccination was carried out by injecting 1 ml Rabisin® subcutaneously along the dorsum using a 3 ml syringe and a 23-gauge needle (Figure 2).

Between Aug 2015 and Jan 2016, 5 ml blood samples were collected from the saphenous vein of the captured animals, placed into an icebox, and transferred to Ocean Park's Clinical Laboratory to assess the antibody titer of the vaccinated macaques (research permit number: AFGRCON 09/50Pt21). Within 12 h of collection, samples were processed, and the extracted sera were stored at −80°C until they could be evaluated. Each macaque was identified uniquely with an interscapular subcutaneous passive integrated transponders (PIT) tag (Avid Identification Systems, CA, USA), corresponding to the collected blood samples, as well as recorded variables such as sex (male or female), age group (subadult or adult), the dates of capture, sampling, and vaccination.

In July 2021, all frozen sera from vaccinated animals were thawed, prepared, and tested for antibodies against rabies virus using a commercial enzyme-linked immunosorbent assay (ELISA) kit, following the manufacturer's instructions (BioPro Rabies ELISA Ab kit). The test kit was a blocking-ELISA for the detection of rabies virus antibodies in serum, plasma, or body fluids. All samples were run in duplicates, and the average of the two optical densities was reported for each sample. The percentage of blocking (PB) was determined for each sample by the following formula: [(OD_NC_-OD_Sample_)/(OD_NC_-OD_PC_)] × 100, where OD_NC_, OD_PC_, and OD_Sample_ were the mean optical densities of the negative control, the positive control, and the macaque serum sample, respectively. The ELISA kit was initially developed and validated to detect rabies antibodies in domestic and wild carnivores. The manufacturers recommend using a cut-off point of PB = 40% for fox sera. The same cut-off value was applied to our macaque samples, considering PB ≥ 40% positive for rabies antibodies.

To assess the potential association between the post-vaccination period (defined as the time period between the 1^st^ vaccination and blood sampling) and rabies antibody titer (PB) in animals that had received one dose of the vaccine, a scatter plot was generated, and Pearson correlation coefficient was estimated. All statistical analyses were performed using Stata v17 (StataCorp LLC, College Station, TX, USA).

## Results

Sixty-five blood samples from the vaccinated macaques were available for inclusion in our final dataset; 53 females and 12 males-62 adults and three subadults. All of these animals had received at least one dose of vaccine (1^st^ vaccination) sometime between March 2008 and January 2016. The post-vaccination period ranged from 21 to 2,779 days. Five of the 65 macaques had a second vaccination record at the time of the blood collection. These five animals had high antibody levels (PB > 74%). Among the remaining macaques that received only one dose of the vaccine, 77% (46/60) were positive for rabies antibodies. The macaques in Hong Kong have been surveyed year-round, 5 days per week, for population studies and monitoring their potential adverse responses to trapping, sterilization, and rabies vaccination. No adverse reactions were noted following the vaccinations.

**Figure 3:**
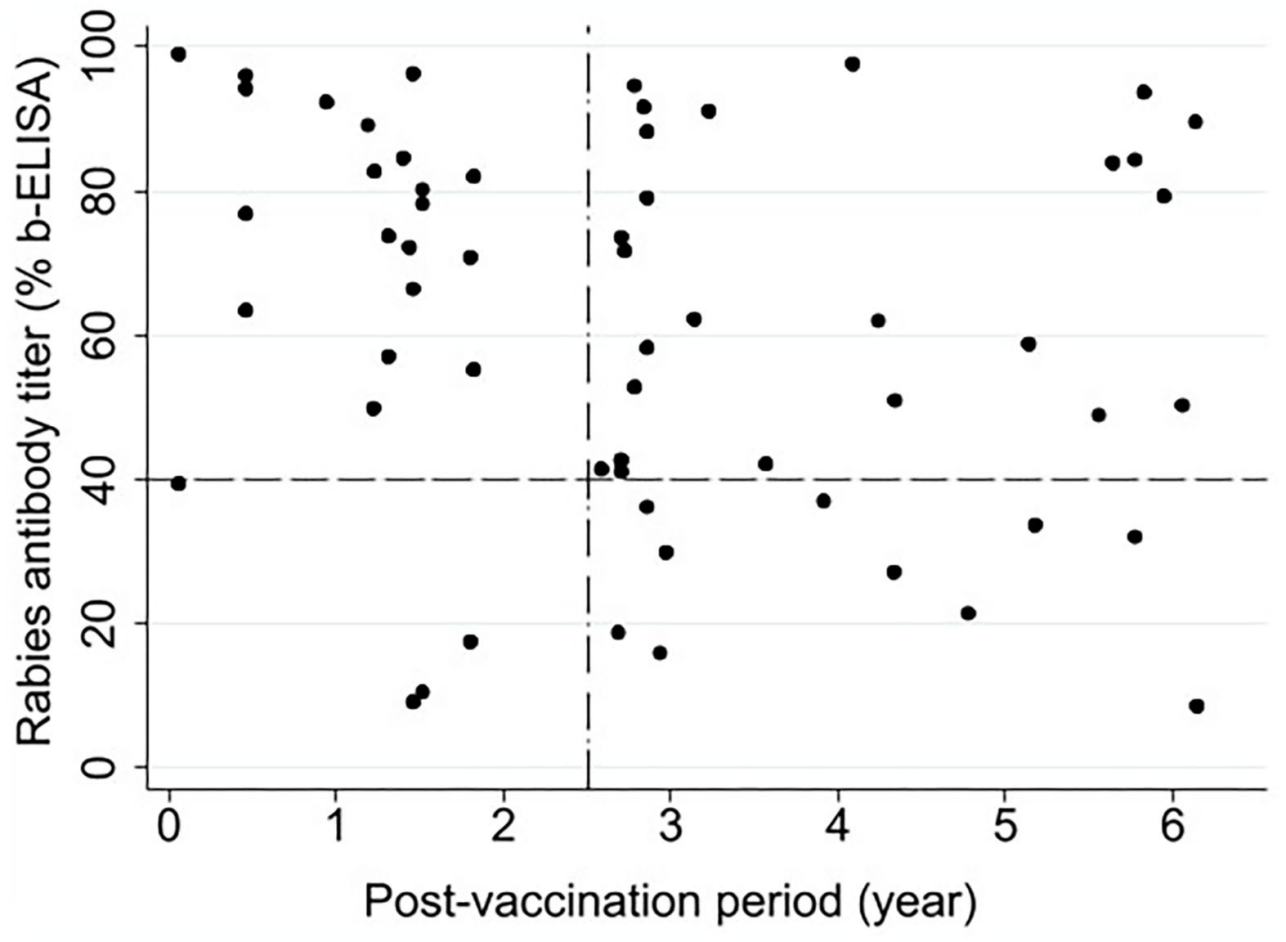
Association between post-vaccination time (year) and rabies antibody titer (% blocking-ELISA) in Hong Kong macaques that received a single dose of Rabisin® rabies vaccine between 2008 and 2016 (*n* = 60). The horizontal reference line at 40% represents the cut-off above which sera were considered positive for rabies virus antibodies. The vertical reference line represents the 2.5-year interval recommended for a booster in the current governmental vaccination strategy.

Figure 3 illustrates the relationship between the post-vaccination period and rabies antibody titer in the macaques that had received a single-dose vaccine (*n* = 60) at the time of sample collection. Only two animals were at 21 days post-vaccination when tested, one with PB = 98.9 and the other with PB = 39.5 (very close to the cut-off point). With respect to the recommended 2.5-year vaccination interval by the government (Figure 3), 87% (21/24) of the animals tested within 2.5 years of their first vaccination and 72% (26/36) of the animals tested beyond this period were positive for rabies antibodies. As shown in Figure 3, no specific association was observed between the post-vaccination period and the antibody titer. Pearson correlation coefficient was −0.18 (*P* = 0.152), indicating no statistically significant linear correlation between the two variables.

## Discussion

Rabisin® (or Imrab®) is an inactivated, adjuvanted rabies vaccine licensed for the immunization of dogs, cats, horses, cattle, sheep, and ferrets by subcutaneous or intramuscular injection. Nonetheless, the vaccine has also been used for immunization in other animal species (15) and is widely used in zoo animals and wild species. There are several reports on the response of macaques to new-generation vaccines for rabies, such as DNA and recombinant vaccines (16–18), and using other viral vectors for rabies virus antigens (19). However, we only found one publication evaluating the efficacy of a commercial inactivated vaccine Defensor® (SmithKline Beecham, West Chester, PA) in macaques (12). The latter study was conducted on seven juvenile pigtail macaques in an experimental setting and concluded that one dose (1 ml) of Defensor® was sufficient to induce high levels of rabies antibodies in the animals, and they recommended annual boosters because of their local circumstances.

A decreasing trend in rabies antibody titers is commonly observed after 1 to 2 years of primary rabies vaccination in humans and animals (20). In our study, 72% of the animals still had positive rabies antibody titers 2.5–6 years post-vaccination, suggesting that the immunity from a single dose of the vaccine may last beyond the recommended threshold for a booster shot by the government. These findings are informative and encouraging for the wildlife conservation and public health agencies in Hong Kong and other countries/regions with similar ecosystems and circumstances to design and adjust their vaccination campaigns at appropriate booster intervals.

According to our ELISA test, a serum sample with PB ≥ 70 is considered to have antibody levels ≥ 0.5 IU/ml based on the Fluorescent Antibody Virus Neutralization test, which is recommended as being protective in humans (21). This was the first time that this ELISA was used for testing sera from macaques. The World Organization for Animal Health (OIE) recommends using ELISA methods for monitoring vaccination campaigns in wildlife populations, “provided the kit used has been validated for the wildlife species under study” (22). One of the limitations of this study is that our test was not validated for macaques. Assuming the application of the test and thresholds are comparable with fox sera, our results suggest 87% of the animals tested within 2.5 years of their first vaccination had positive levels of antibodies. Overall, 77% of all single-vaccinated animals had levels of immunity to the virus (i.e., PB > 40). The low levels of antibodies in the three animals vaccinated within 2.5 years could be attributed to various reasons, such as a natural range of immune responses to vaccination in animals and age differences (20, 23).

The current rabies vaccination practice in the macaque population of Hong Kong (including annual trap and vaccination of captured animals and providing booster shots to those animals that received the vaccine longer than 2.5 years) elicited rabies antibodies in a high proportion of animals (overall, 51/65) over a long period of time. However, whether these levels would be protective against a natural challenge with the virus cannot be affirmed without further investigation. Our findings support not only the currently recommended vaccination interval but also the possibility of extending the interval between vaccination and booster shots in order to reduce the labor and costs associated with the vaccination program. Our final goal is to provide a minimum level of herd immunity in the macaque population to prevent the potential spread of the disease and resultant mass culling policies if rabies occurs in macaques or other sympatric species. The latter highlights the need for further studies on population density, vaccination proportion, and herd-immunity models.

## Data Availability Statement

The raw data supporting the conclusions of this article will be made available by the authors, without undue reservation.

## Ethics Statement

The animal study was reviewed and approved by Agriculture, Fisheries and Conservation Department of the Government of the Hong Kong SAR (AFCD), according to the guidelines for animal handling. A special permit for the collection of blood samples was obtained from AFCD, under Cap 170 and Rabies Ordinance, Cap 421.

## Author Contributions

PM led the project and contributed to the writing of the original manuscript. ON conducted the statistical analyses and wrote the initial draft of the manuscript. SS contributed to the analysis and added expert inputs to the original manuscript. IM reviewed and added expert inputs to the original manuscript. W-SH processed the samples and conducted the laboratory work. KK coordinated the sample collection and project. All authors further reviewed, edited, and approved the final manuscript.

## Conflict of Interest

PM, W-SH, and KK are employed by Ocean Park Corporation Hong Kong. The remaining authors declare that the research was conducted in the absence of any commercial or financial relationships that could be construed as a potential conflict of interest.

## Publisher's Note

All claims expressed in this article are solely those of the authors and do not necessarily represent those of their affiliated organizations, or those of the publisher, the editors and the reviewers. Any product that may be evaluated in this article, or claim that may be made by its manufacturer, is not guaranteed or endorsed by the publisher.
